# The Clinical Association between Carbon Monoxide Poisoning and Myocardial Injury as Measured by Elevated Troponin I Levels

**DOI:** 10.3390/jcm12175529

**Published:** 2023-08-25

**Authors:** Bhakti Patel, Jideofor Omeh, Gary Tackling, Rohun Gupta, Tiffany Sahadeo, Viliane Villcant, Tashina Dussie, Mirette Atnas, Ofek Hai, Roman Zeltser, Amgad N. Makaryus

**Affiliations:** 1 Department of Cardiology, Donald and Barbara Zucker School of Medicine at Hofstra/Northwell, Hempstead, NY 11549, USA; 2 Department of Cardiology, Nassau University Medical Center, East Meadow, NY 11554, USA

**Keywords:** carbon monoxide poisoning, myocardial injury, elevated troponin

## Abstract

Carbon monoxide (CO) poisoning accounts for over 50,000 estimated emergency room visits and approximately 1200 deaths per year in the US. Despite the high prevalence, there is a paucity of data looking at the association between laboratory biomarkers and clinical outcomes. Our study investigates the association between myocardial injury as assessed by increased troponin levels and its effect on in-hospital outcomes in CO poisoning. A total of 900 sequential charts of patients presenting with CO poisoning between 1 January 2012, and 31 August 2019, at our tertiary center with regional hyperbaric chamber and burn unit, were reviewed. Of the 900, a total of 488 patients had elevated carboxyhemoglobin levels. Of these 488 patients, 119 (24.4%) also had blood troponin levels measured. Patients were stratified based on the presence or absence of myocardial injury as evidenced by highly sensitive serum troponin I (TnI) level > 0.5 ng/mL to determine if a correlation exists relating to myocardial injury and risk of major adverse events. Mean age was 51.2 years, 58.8% were males, 35.3% were non-White, and 10.1% were intentional CO poisonings. Comorbidities included hypertension: 37%, diabetes: 21%, smoking: 21%, hyperlipidemia: 17.6%, coronary artery disease: 11.8%, asthma: 5.9%, heart failure: 5%, atrial fibrillation: 4.2%, and chronic obstructive pulmonary disease: 4.2%. Myocardial injury occurred in 22 patients (18.5%) and was associated with increased likelihood of requiring intensive care admission (54.5% vs. 20.6%, *p* = 0.002) and intubation (40.9% vs. 14.4%, *p* = 0.008). TnI elevation was associated with higher in-hospital mortality (*p* = 0.008, OR 21.3) compared to patients without TnI elevation. Older age was independently associated with increased in-hospital mortality (*p* = 0.03, OR 1.08). When controlling for age, in-hospital mortality remained statistically significant (*p* = 0.01, OR 21.37). No significant difference was found with respect to age, comorbidities, gender, race, ethnicity, or hospital length of stay in patients with and without myocardial injury. Myocardial injury induced by CO exposure occurs frequently and adversely affects clinical outcomes. Further research is needed to help guide physicians in the management of CO poisoning and associated myocardial injury to improve patient outcomes.

## 1. Introduction

Carbon monoxide (CO) poisoning is the reason for over 50,000 estimated emergency room visits and approximately 1200 deaths per year in the United States [1]. It is also the leading cause of death by poisoning [2]. Carbon monoxide is a colorless, odorless, and tasteless gas that is often referred to as the silent killer because of the inability to detect it without proper equipment. Essentially, carbon monoxide is a gas that is found in any combustion source, including automobile exhaust, industrial solvents, fire, and faulty furnaces. It is produced by the incomplete combustion of fossil fuels like coal, oil, and gas [3]. The risk of CO poisoning increases as CO accumulates from these sources in an enclosed area. Symptoms of poisoning present as headache, dizziness, weakness, nausea, vomiting, chest pain, and confusion [4]. Moreover, a common cardiovascular manifestation of moderate to severe CO poisoning is myocardial injury [5]. Myocardial injury is defined as damage to the heart muscle, leading to a variety of cardiovascular complications. The spectrum of myocardial involvement includes damage at the cellular or subcellular level. Additionally, it clinically involves cardiomyopathy, heart failure, angina attack, myocardial infarction, arrhythmias such as ventricular premature beats and atrial fibrillation, bradycardia, atrioventricular block, cardiogenic shock, and sudden death [6]. Other cardiovascular complications of carbon monoxide poisoning include myocardial stunning, left ventricular dysfunction, pulmonary edema, and arrythmias [7]. Importantly, the extent of carbon monoxide poisoning does not proportionally equal the degree of myocardial damage and high troponin levels. Patients with myocardial injury can commonly present with shortness of breath as the main symptom along with chest tightness [8].

Myocardial injury from CO poisoning ensues as a result of cellular damage and tissue hypoxia. Particularly, CO has a 210-times-higher affinity for hemoglobin than oxygen [9]. When carbon monoxide is introduced into the body, it binds to hemoglobin, forming carboxyhemoglobin (COHb). Carbon monoxide outcompetes oxygen and causes oxygen saturation of hemoglobin to decrease, leading to a reduction in oxygen delivery and tissue hypoxia. The organ systems that are most sensitive to hypoxia are those with a large oxygen demand, such as the heart. This result of myocardial injury and dysfunction is evidenced by an increase in troponin I (TnI) levels, ECG abnormalities, and myocardial necrosis [10]. Myocardial damage leads to an increase in cardiac troponins in blood circulation as they function as regulatory proteins within the myocardium. On the actin filament, the three-unit troponin complex (troponin I, T, and C), as well as tropomyosin, is located and functions as a calcium regulation for cardiac muscle contraction [11]. Additionally, CO can directly affect the cardiovascular system, causing vasodilation, or widening of blood vessels. This can lead to a drop in blood pressure which decreases perfusion to vital organs. A decrease in perfusion to the heart can cause myocardial injury by depriving the heart muscle of oxygen and nutrients [12]. Lastly, CO can also cause inflammation and oxidative stress in the cardiovascular system. It activates inflammatory pathways and increases the presence of reactive oxygen species that damage cells [13]. An environment of oxidative stress and inflammation promote atherosclerosis via the buildup of plaques in arteries, which then further decreases blood flow and perfusion to the heart to exacerbate myocardial injury. Although there is a high prevalence of myocardial injury and CO poisoning, there are insufficient data that examine the association between laboratory biomarkers and clinical outcomes in these patients. Our study investigates the association between troponin elevations and their effect on clinical outcomes in carbon monoxide poisoning patients.

## 2. Materials and Methods

The design of this study is a retrospective cohort study. Electronic medical records were reviewed and evaluated with a total of 900 sequential charts of patients presenting with carbon monoxide exposure between the dates of 1 January 2012, and 31 August 2019 at Nassau University Medical Center, which is our tertiary center that has a regional hyperbaric chamber and burn unit. CO poisoning was defined as a carboxyhemoglobin (COHb) level > 10% [14]. Patients who met this criterion of CO poisoning were then selected and evaluated for whether they would have troponin I (TnI) levels tested as well. It was imperative to evaluate for presence of both COHb and TnI levels in each patient to understand the extent of myocardial injury following CO poisoning. Of the 900 patient charts reviewed, a total of 488 patients had elevated carbon monoxide levels. Of these 488 patients, 119 (24.4%) patients had troponins drawn as well. The patients were then stratified based on the presence or absence of myocardial injury, as evidenced by highly sensitive serum troponin I levels > 0.5 ng/mL, to determine if a correlation existed relating to myocardial injury and risk of major adverse events. Data were gathered for analysis, specifically including demographics such as age, gender, and race. Patients were further analyzed for the presence of traditional pre-existing cardiac conditions, such as hypertension (HTN), coronary artery disease (CAD), advanced age, and smoking status. A multivariate analysis via SPSS^®^ analytic software was performed to determine correlations and associations, with a *p* value less than 0.05 considered as significant.

## 3. Results

The study population was selected by evaluating every patient that presented with carbon monoxide exposure first as we sequentially discovered all the patients from our medical record system upon review for 1 January 2012 to 31 August 2019. Baseline demographic and clinical characteristics of the study population are shown in Table 1. There were 58.8% men and 41.2% women in this study. The mean age of the patients was 51.2 years and the majority of cases (90%) were due to unintentional/accidental exposure to CO. Patient comorbidities were also analyzed and assessed as possible confounding variables between patient groups for cardiovascular complications that can lead to myocardial injury. Comorbidities of the patients included HTN—37%, diabetes (DM)—21%, smoking—21%, hyperlipidemia—17.6%, CAD—11.8%, asthma—5.9%, heart failure (CHF)—5%, atrial fibrillation—4.2%, and chronic obstructive pulmonary disease (COPD)—4.2%. However, baseline comorbidities did not show significant difference between groups. The comparison groups involved patients with troponin I elevation versus patients without troponin I elevation. It was found that an increase in age was associated with higher likelihood of in-hospital mortality (OR 1.08, *p* = 0.03). When adjusted for age, a higher mortality of these patients was noticed secondary to myocardial injury. This association between mortality and myocardial injury remained statistically significant (OR 21.37, *p* = 0.01). No statistically significant difference was found with respect to comorbidities, gender, race, or ethnicity in patients with and without myocardial injury.

**Figure 1:**
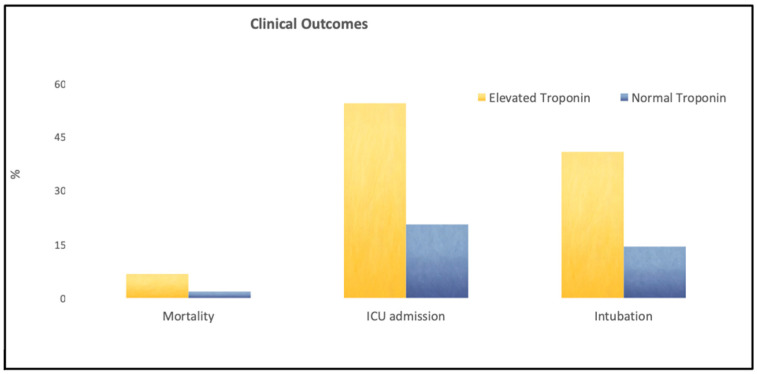
Myocardial injury induced by CO exposure adversely impacts clinical outcomes. This figure compares the percentage of patients with elevated troponin versus normal troponin who developed complications such as mortality, ICU admission, and intubation.

Within our study population, myocardial injury, determined by elevated troponin levels, occurred in 22 patients (18.5%). The association of myocardial injury induced by CO exposure and clinical outcomes is shown in Figure 1. Myocardial injury was associated with a higher in-hospital mortality (OR 21.3, *p* = 0.008). Patients with TnI elevation were more likely to require intensive care unit (ICU) admission (54.5% vs. 20.6%, *p* = 0.002) and intubation (40.9% vs. 14.4%, *p* = 0.008) compared to patients without TnI elevation.

## 4. Discussion 

Myocardial injury can be seen as a consequence of moderate to severe carbon monoxide poisoning. As described earlier, carbon monoxide can lead to a decrease in blood perfusion via inflammation, decreased hemoglobin levels to carry oxygen, and exacerbation of atherosclerosis. This decrease in perfusion impacts blood supply to the heart. Carbon monoxide binds to hemoglobin at a 200- to 250-times-higher affinity than oxygen such that carbon monoxide exposure, even if it is low, can result in decreased oxygen delivery and increased tissue hypoxia [15]. The ischemia and neurological injury caused by these effects leads to injury to the myocardium. Essentially, myocardial ischemia leads to an increased release of the inflammatory cascade involving leukocytes, which further damages functional tissue leading to myocardial injury [16]. Myocardial toxicity from carbon monoxide exposure has been associated with an increase in short- and long-term mortality [17]. Upon presentation of carbon monoxide poisoning to the emergency department, a study by Yurtseven et al. found 49 of the 163 patients in the study population to have normal carboxyhemoglobin levels. Troponin I levels were found to be elevated in 26 patients, with a mean value of 0.38 ng/mL. However, in Yurtseven et al.’s study, the majority of patients were poisoned by stoves (81.9%), then by hot water boilers (10.5%) and fires (5.8%). This study reinforced that troponin I levels can be seen to rise in carbon monoxide intoxication. It is important to note that although elevated carboxyhemoglobin confirms the diagnosis of carbon monoxide intoxication, normal levels do not rule this diagnosis out [18]. 

In this study, we report on the positive association between myocardial injury, as evidenced by a release of troponin levels into the bloodstream, and an increase in the in-hospital mortality as a result of myocardial injury, ICU admissions, and number of patients intubated for respiratory compromise. The presence of myocardial injury is known to be associated with a poor prognosis. In a 2006 study by Henry et al., myocardial injury was found to be a significant predictor of long-term mortality. Specifically, among the 37% who suffered myocardial injury from the 230 total carbon monoxide poisoning patients, 38% eventually died, compared to 15% of patients who did not sustain a myocardial injury (*p* = 0.009) [13]. However, initial carbon monoxide poisoning was intentional (suicide attempt) in 40% of the study population, which predominately consisted of Caucasian males [15]. Our study was primarily conducted on patients with accidental exposure (90%). Many of these patients were exposed following Hurricane Sandy (a devastating hurricane that struck our area of Long Island, New York, in October 2012), faulty heating devices, or smoke inhalation due to house fires. Additionally, our study’s population included 41.2% females. Kao et al. reported that myocardial injury independently predicted a poor short-term outcome, defined as an in-hospital death or neurologic sequelae at discharge, in patients with severe carbon monoxide poisoning who had received hyperbaric oxygen therapy. A total of 9 patients died while in the hospital, 32 patients had neurological complications at the time of discharge, and a 50.6% incidence of poor outcome was determined [19]. However, prolonged lag time from the end of carbon monoxide exposure to arrival at the emergency department and start of treatment played a role in these poor outcomes as well [19]. A retrospective study by Huysal et al. included 141 patients with acute carbon monoxide intoxication. After dividing the patients into three groups based on the carboxyhemoglobin level, they found even mild carbon monoxide poisoning (COHb level < 15%) to be associated with quantifiable circulating levels of Troponin T [20]. 

Furthermore, our study also determined that patients with elevated troponin I levels were more likely to need ICU admission and intubation. This is consistent with studies involving different disease states. Sepsis is the leading cause of death and in-hospital mortality throughout the world [21]. In ICU patients admitted with sepsis, troponin levels were associated with a longer duration of mechanical ventilation and intubation [22]. A retrospective study by Alatassi et al. found elevated troponin-I levels observed in medical-surgical critically ill patients with a level-dependent association with hospital mortality [23]. With ICU admission after carbon monoxide poisoning, there is also an increase in the likelihood of patient mortality. According to Liao et al., a retrospective study identified 17.8% of 787 patients with carbon monoxide poisoning admitted to the ICU. The mortality rate was found to be 14.3% among these ICU patients. Specifically, the study determined that an increased mortality risk in ICU patients for this population was a result of dysfunction of more than three organ systems, a Glasgow Coma Scale score of 3, and Acute Physiology and Chronic Health Evaluation II score greater than or equal to 25 [7]. 

Prior studies have described myocardial injury as a cardiovascular manifestation of carbon monoxide poisoning. A study by Dragelyte et al. stated that carbon monoxide poisoning can lead to angina and myocardial injury in patients of a younger age irrespective of existing cardiovascular comorbidities [24]. At any extent of carbon monoxide poisoning level, myocardial injury can ensue as a transient, reversible, or permanent presentation [25]. In a study by Satran et al., 37% of carbon monoxide poisoning patients were found to have myocardial injury, as determined via ECG or biomarkers (troponin I level of > 0.7 ng/mL or CK-MB mass > 5.0 ng/mL). The study population in this case had a mean age of 47.2 years with 72% men [5]. Garg et al. reported 35% of acute carbon monoxide poisoning patients with a 5% in-house mortality rate having elevated biomarkers of CK-MB and troponin I as an indication of myocardial injury [17]. In contrast, the prevalence of myocardial injury of 18.5% in our study could be attributed to the exclusion of CK-MB from the definition of myocardial injury in our population because of its lower specificity for myocardial cellular injury than cardiac troponins [26].

An important indicator of myocardial injury is elevated troponin levels. Troponin assays were found to be more sensitive and more specific than CK-MB assays [26]. About 33% of patients presenting with symptoms of acute coronary syndromes were found to have elevated troponin levels in the absence of elevated CK-MB levels [11]. CK-MB is found outside of the heart, included in the skeletal muscle, gastrointestinal tract, and uterus of pregnant women [27]. Peak troponin I levels in CO poisoning patients who developed myocardial injury were found to occur at 11 h after exposure and normalized by 65 h [28]. In our study, we particularly used troponin I > 0.5 ng/mL to conclude a myocardial injury occurrence. Leman et al. reported high troponin levels to be beneficial in predicting clinical outcomes in children with carbon monoxide poisoning. A total of 331 pediatric patients with carbon monoxide poisoning were included in the study, of which 51 were admitted to the pediatric intensive care unit and 6 died. This study found risk factors of a high troponin T level at presentation as well as a low Glasgow Coma Scale score and high leukocyte count [29]. In contrast, our study includes a study population of only adults with CO poisoning.

The clinical implications of myocardial injury in terms of hospital mortality are profound. Patients with myocardial injury require careful monitoring, prompt identification of underlying causes, and tailored therapeutic interventions. Clinical application of this study’s findings can be implemented in how physicians approach patients with carbon monoxide poisoning and elevated troponin I levels on initial presentation. Physicians should screen for myocardial injury to diagnose this early and begin treatment in an effort to prevent a decline in the patient’s health course from prolonged myocardial injury. Aggressive management of myocardial injury involves optimal medical therapy, revascularization strategies, and targeted interventions to mitigate inflammation and oxidative stress surrounding the heart. This allows for an increased potential for patient outcome improvement. With improvement of patient outcomes and early recognition of myocardial injury leading to early treatment of the diagnosis, there will be a reduction in further complications such as decreased mortality rates.

Future research direction is needed to understand and identify the onset of myocardial injury for early management of a patient presenting with carbon monoxide poisoning. Specifically, future research should focus on refining risk stratification models, investigating novel therapeutic approaches, and exploring the long-term impact of myocardial injury on patient prognosis. Furthermore, further research should separately evaluate the association between carbon monoxide poisoning and myocardial injury in patients with coronary heart diseases. At a baseline, patients with coronary artery disease have a decreased blood and oxygen supply to the myocardium. An increase in cholesterol deposition in blood vessels leads to narrowing of the arteries, which thus decreases blood flow to the heart [30]. Thus, patients with coronary heart disease are more susceptible to myocardial injury as the oxygen supply is further compromised during carbon monoxide poisoning. Our study compared the association between carbon monoxide poisoning and myocardial injury in patients with and without elevated troponin I levels who had no significant differences in baseline comorbidities, such as coronary artery disease.

While this study on myocardial injury and carbon monoxide poisoning provides valuable insights into the association between these two conditions and further long-term sequelae, it is essential to acknowledge several potential limitations to our study. This is a retrospective cohort study from a single center with a relatively small number of patients matching our inclusion criteria. As a result, the statistical power and generalizability of the results from this study population can be limiting. Additionally, retrospective studies can be more prone to recall bias or incomplete data follow-up. Moreover, outcomes are related to in-hospital/peri-hospitalization measures, and further long-term effects beyond hospitalization were not measured in our study. 

## 5. Conclusions

Our study demonstrates that myocardial injury induced by carbon monoxide poisoning occurs frequently and adversely affects clinical outcomes. Essentially, our study population identified carbon monoxide poisoning patients by carboxyhemoglobin levels, and myocardial injury by elevated troponin I levels. Imperatively, this study serves as a significant predictor of adverse outcomes and requires attention from healthcare providers to optimize patient management. We found an increased association between myocardial injury from carbon monoxide poisoning and in-hospital mortality, ICU admission, and number of patients intubated for respiratory compromise. Despite our data showing the significant increased risk of downstream cardiovascular events in CO poisoning patients presenting with elevated troponin, little emphasis has been placed in the medical field on this area, and in guideline statements, on appropriate follow-up cardiac care in patients with carbon monoxide exposure and poisoning. Further research is needed to help guide physicians in the management of carbon monoxide exposure/poisoning and myocardial injury to improve patient outcomes.

## Figures and Tables

**Table 1:** Patient demographics and clinical risk factors.

Demographics	Race	Comorbidities
Male 58.8%	White 64.1%	Hypertension 37.0%
Female 41.2%	Black 21.0%	Diabetes 21.0%
	Asian 5.0%	Hyperlipidemia 17.6%
	Hispanic 9.2%	CAD 11.8%
	Other/Unknown 0.7%	CHF 5.0%
		Afib 4.2%
		COPD 4.2%
		Asthma 5.9%
		Smoker 21%

## Data Availability

Pertinent data is contained within the article.
